# Dendritic Cells Cause Bone Lesions in a New Mouse Model of Histiocytosis

**DOI:** 10.1371/journal.pone.0133917

**Published:** 2015-08-06

**Authors:** Frédéric Grosjean, Sonia Nasi, Pascal Schneider, Véronique Chobaz, Alexandra Liu, Vanessa Mordasini, Kristell Moullec, Paolo Vezzoni, Christine Lavanchy, Nathalie Busso, Hans Acha-Orbea, Driss Ehirchiou

**Affiliations:** 1 Department of Biochemistry CIIL, University of Lausanne, Epalinges, Switzerland; 2 DAL, Service of Rheumatology, Laboratory of Rheumatology, University of Lausanne, CHUV, Epalinges, Switzerland; 3 UOS/IRGB, Milan Unit, CNR, via Fantoli 15/16–20138 Milano, and Humanitas Research Center, Rozzano Italy; Université de Lyon - Université Jean Monnet, FRANCE

## Abstract

Langerhans cell histiocytosis (LCH) is a rare disease caused by the clonal accumulation of dendritic Langerhans cells, which is often accompanied by osteolytic lesions. It has been reported that osteoclast-like cells play a major role in the pathogenic bone destruction seen in patients with LCH and these cells are postulated to originate from the fusion of DCs. However, due to the lack of reliable animal models the pathogenesis of LCH is still poorly understood. In this study, we have established a mouse model of histiocytosis- recapitulating human disease for osteolytic lesions seen in LCH patients. At 12 weeks after birth, severe bone lesions were observed in our multisystem histiocytosis (Mushi) model, when CD8α conventional dendritic cells (DCs) are transformed (MuTuDC) and accumulate. Most importantly, our study demonstrates that bone loss in LCH can be accounted for the transdifferentiation of MuTuDCs into functional osteoclasts both in vivo and in vitro. Moreover, we have shown that injected MuTuDCs reverse the osteopetrotic phenotype of oc/oc mice in vivo. In conclusion, our results support a crucial role of DCs in bone lesions in histiocytosis patients. Furthermore, our new model of LCH based on adoptive transfer of MuTuDC lines, leading to bone lesions within 1–2 weeks, will be an important tool for investigating the pathophysiology of this disease and ultimately for evaluating the potential of anti-resorptive drugs for the treatment of bone lesions.

## Introduction

Langerhans cell histiocytosis (LCH) is a rare disease caused by the clonal accumulation of dendritic Langerhans cells. LCH is characterized by skin rashes, lymphadenopathy and splenomegaly but can also become a multi-systemic disease and in most cases the bone presents an isolated lytic lesion [1,2].

It has been reported that osteoclast-like cells play a major role in the pathogenic bone destruction seen in patients with LCH [3,4]. Indeed, these cells expressed the characteristic osteoclast markers such as tartrate-resistant acid phosphatase (TRAP) as well as the enzymes Cathepsin-K and matrix metalloproteinase-9. Coury et al. have demonstrated that the osteoclast-like cells originate from the fusion of dendritic cells (DCs), the major cell type found in LCH lesions through an IL-17-dependent pathway [3].

DCs are known to be specialized mononucleated antigen-presenting cells, able to both initiate immune responses and to induce tolerance [5]. Recent studies have suggested that DCs display a high developmental and functional plasticity depending on local factors and stimuli encountered during their differentiation and maturation. In fact, it has been shown that immature DCs can be transdifferentiated into osteoclats, the bone-resorbing multinucleated giant cells, when cultured with osteoclastogenic factors, M-CSF, RANKL and IL-17 [6–9].

Despite the improved knowledge on LCH, the factors and pathogenesis that define this disease are still poorly understood. A major obstacle to our understanding of the LCH pathology is the inability to conduct prospective and well-controlled clinical studies due to the lack of reliable animal models [10].

An animal model for histiocytosis has been established by infecting mice with the malignant histiocytosis sarcoma virus [10–12] and recently, in our lab, a mouse model of multisystem histiocytosis was developed by transgenically expressing the SV40 T oncogene in DCs using a CD11c-promoter construct [13]. In these mice between 3 and 4 months after birth, conventional Langerin-expressing CD8α DCs transform and infiltrate bone marrow, spleen liver, thymus and mesenteric lymph nodes. The transformed DCs maintain all the functions of normal DCs [13,14].

Animal models for LCH are urgently needed to assess the pathologic features and to provide insights regarding the etiology of osteolytic lesions. In this study, we report the characterization of the first mouse model of histiocytosis-like disease for osteolytic lesions.

## Materials and Methods

### Mice

The Mushi Transgenic (Tg) mice (CD11c:SV40LgT-transgenic C57BL/6 mice) were developed in our lab by transgenically expressing the SV40 T oncogene and GFP in DCs using a CD11c-promoter construct [13]. In Mushi Tg mice between 3 and 4 months after birth, conventional Langerin-expressing CD8α DC transform and massively infiltrate bone marrow, spleen liver, thymus and mesenteric lymph nodes. C57BL/6 mice were purchased from Harlan. (C57BL/6JxB6C3Fe-*a*/*a*-Tcirg1oc/J) *oc*/+ mice were purchased from the Jackson Laboratory, CD3ε KO (B6;CBA-Tg(CD3E)26Cpt/J, Jackson), rag-2 KO (B6(Cg)-*Rag2* ^*tm1*.*1Cgn*^/J, Jackson) are described in [15,16]. Experiments were approved and controlled by the Swiss federal and cantonal veterinary authorities. The mice were sacrificed by CO2 inhalation for sample collection.

### DC line cells culture

The DC line cells (MuTuDCs) used at low passage number originate from spleen tumors of Mushi Tg mice. The MutuDC lines were derived as described in [14]. The cells were kept in culture at 37°C in a humidified incubator with 5% CO2, in IMDM-glutamax (Lifetechnologies) supplemented with 10% heat inactivated FCS, 10 mM Hepes, 50 μM β-mercaptoethanol, 50 U/mL of penicillin and 50 μg/mL streptomycin. MuTuDCs were harvested after 5 min incubation with 5 mM EDTA-containing cell dissociation buffer.

### Flow Cytometry

The antibodies used were specific to CD11c (clone N418, PECy7, eBioscience), CD45R-B220 (clone RA3-6B2, Pacific-Blue, BioLegend), CD8α (clone 54–6.7, FITC, eBioscience or PeCy5.5, BioLegend), CD11b (clone M1/70, APC, eBioscience) and MHC-II (clone M5/114.15.2, PE, Pharmingen). Flow cytometric acquisitions were performed using a LSR II cytometer (Becton Dickinson). Data processing and analyses were done with FACSDiva (version 6.1.3, Becton Dickinson) and FlowJo (version 9, Treestar Inc.) softwares.

### Preparation of bones

The mouse skull, femur and tibia were removed and cleaned from adherent tissue by overnight incubation at 56°C in proteinase K buffer (proteinase K 1:80 in TEN buffer). Bones were then washed once with 70% EtOH and left to dry for several hours. The bone lesions were identified using fluorescent stereomicroscopy (Leica, M205FA).

### RNA isolation and cDNA synthesis

Total RNA was isolated from MuTuDC samples using the ready-to-use reagent for the isolation of total RNA (TRIZOL reagent; Invitrogen Life Technologies). It was then extracted with chloroform, precipitated in propanol, washed twice in ethanol 70%, air-dried, dissolved in RNase-free water and finally quantified by spectrophotometry (Nanodrop ND-1000, ThermoScientific). Synthesis of cDNA was performed using random nonamer primers and the Superscript II Reverse Transcriptase kit (Invitrogen) using 1 μg of total RNA.

### Quantitative real-time PCR

The qRT-PCR was performed using SYBR Green mix on LightCycler480 (384-well plate, 5 μL reaction) from Roche Diagnostics. The following primers were used: TRAP, F 5-CAGGAGACCTTTGAGGACGTG-3, R 5-GTGGAATTTTGAAGCGCAAAC-3; NFATc1, F 5-CCTTCCCACAGCACACTCTG-3, R 5-TAGGCCCAGGTAGGAGGTGA-3; cFos, F 5-GTCAAGAGCATCAGCAACGTG-3 R5-GTAGTGCAGCCCCGGAGTACAG-3; cathepsin K, F5-TGGCTCGGAATAAGAACAACG-3 R 5- GCACCAACGAGAGGAGAAATG-3.

QRT-PCR was performed with at least technical duplicates. For the analysis, expression of each gene was normalized to the housekeeping gene β-actin, F 5-GCACAGCTTCTTTGCAGCTCCTTCG-3, R 5-TTTGCACATGCCGGAGCCGTTG-3 generating a single value per biological replicate. Relative change in mRNA expression was calculated using the qBasePlus software (Biogazelle).

### *In vitro* osteoclast differentiation and TRAP immunohistology

MuTuDCs were seeded at 10^4^ cells/well onto glass culture slides (Becton Dickinson, 354108) in IMDM-glutamax supplemented with 10% fetal calf serum, 100 U/mL penicillin, and 100 μg/mL streptomycin in the presence of 25 ng/mL M-CSF and 100 ng/mL RANK-L and cultured for 12 days. Cytokines (M-CSF, RANK-L) were added at the beginning of the culture and then replenished every 3 days. For immunofluorescence staining of TRAP, cells cultured on glass slide were first incubated with anti-TRAP antibody (H-300, SantaCruz), then treated with the appropriate secondary biotinylated antibody and streptavidin labeled rhodamine. Nuclei were stained with DAPI. Observations were performed by epifluorescence using a Leica confocal microscope.

### Adoptive transfer of MuTuDCs

MuTuDCs (2x10^6^ cells per mouse) were injected either subcutaneously on the calvaria (close to the left parietal bone), intravenously or intraperitoneally (in case of oc/oc mice on day 2 after birth). Since our MuTuDCs express a viral oncogene, they are rejected after adoptive transfer into C57BL/6 mice, the tumor occurs once CD8beta+ T cells are depleted with 250ug of anti-CD8 beta (clone H35) via i.p. injection (first injection 1 day prior to DC transfer and the second 3 days later). The efficiency of depletion is routinely between 95 and 99% in our hands. After 4 weeks, mice were sacrificed and hind limbs and skulls were collected. Osteolysis was monitored either directly post-sacrifice by CT Scan imaging or by stereomicroscopy post-proteinase K digestion.

### Bisphosphonate and mOPG treatments of Mice

One week after subcutaneous injection of MuTuDCs, mice were injected intraperitoneally with a single dose of Aclasta (zoledronic acid, Novartis, 2μg in 200μl of PBS) or subcutaneously with mOPG (mouse OPG-Fc, 200μg in 200μl of PBS) twice per week, close to the site of MuTuDC injection. Mouse OPG-Fc was produced as described for TRAILR2.Fc [17].

### Microcomputed Tomography Analyses (Micro CT)

MicroCT scans of femur, ilium, tibia and skulls were performed using a SkyScan 1076 X-ray μCT scanning system (SkyScan, Kontich, Belgium). The scanning parameters (18 μm resolution, 60 kV, 167 μA, 0.4° rotation step over 360°, 0.5 mm Aluminum filter, 1180 ms exposure time) were kept identical for all the samples, included the two 2-mm diameter phantoms of calcium density 0.25 g/cm^3^ and 0.75 g/cm^3^ (Gloor Instruments, Switzerland) considered as reference. Images were reconstructed using NRecon Version 1.6.6.0 (Skyscan, Kontich, Belgium) with the following standardized parameters for all samples: grey-values = 0.0000–0.105867, Ring Artifact Reduction = 3, Beam Hardening Correction = 40%). Bone analyses were performed using CTAnalyzer Version 1.10 (SkyScan, Kontich, Belgium). The Hounsfield Unit (HU) and Bone Mineral Density (BMD) calibrations were first applied to the dataset using the phantom scans. Manufacturer's HU and BMD calibration procedures were then followed. Different Volumes of Interest (VOIs) were considered for the quantitative analysis of bone mineral density (BMD, g/cm^3^), percent bone volume (BV/TV, %), bone surface density (BS/TV, mm^-1^) and trabecular number (Tb.N, mm^-1^).

### Bone resorption assay

To assess resorption activity, MuTuDCs were seeded in 96-well plates containing dentine slices (Immunodiagnostic Systems) and IMDM-glutamax supplemented with 50 ng/mL RANK-L and 25 ng/mL M-CSF for 12 days in a 5% CO_2_ incubator. Following complete cell removal by immersion in water, dentine slices were stained with 1% toluidine blue in 0.5% sodium tetraborate for 5 minutes. Excess staining was removed by rinsing in tap water and then air-drying. The resorption pits were identified using a fluorescent stereomicroscopy (Leica, M205FA).

### Statistical Analysis

Statistical analyses were performed using the nonparametric Mann-Whitney test. Differences were considered significant for *p<0.05, **p<0.01, ***p<0.001.

## Results

### 1 Bone lesions are found in Mushi transgenic mice

**Fig 1:**
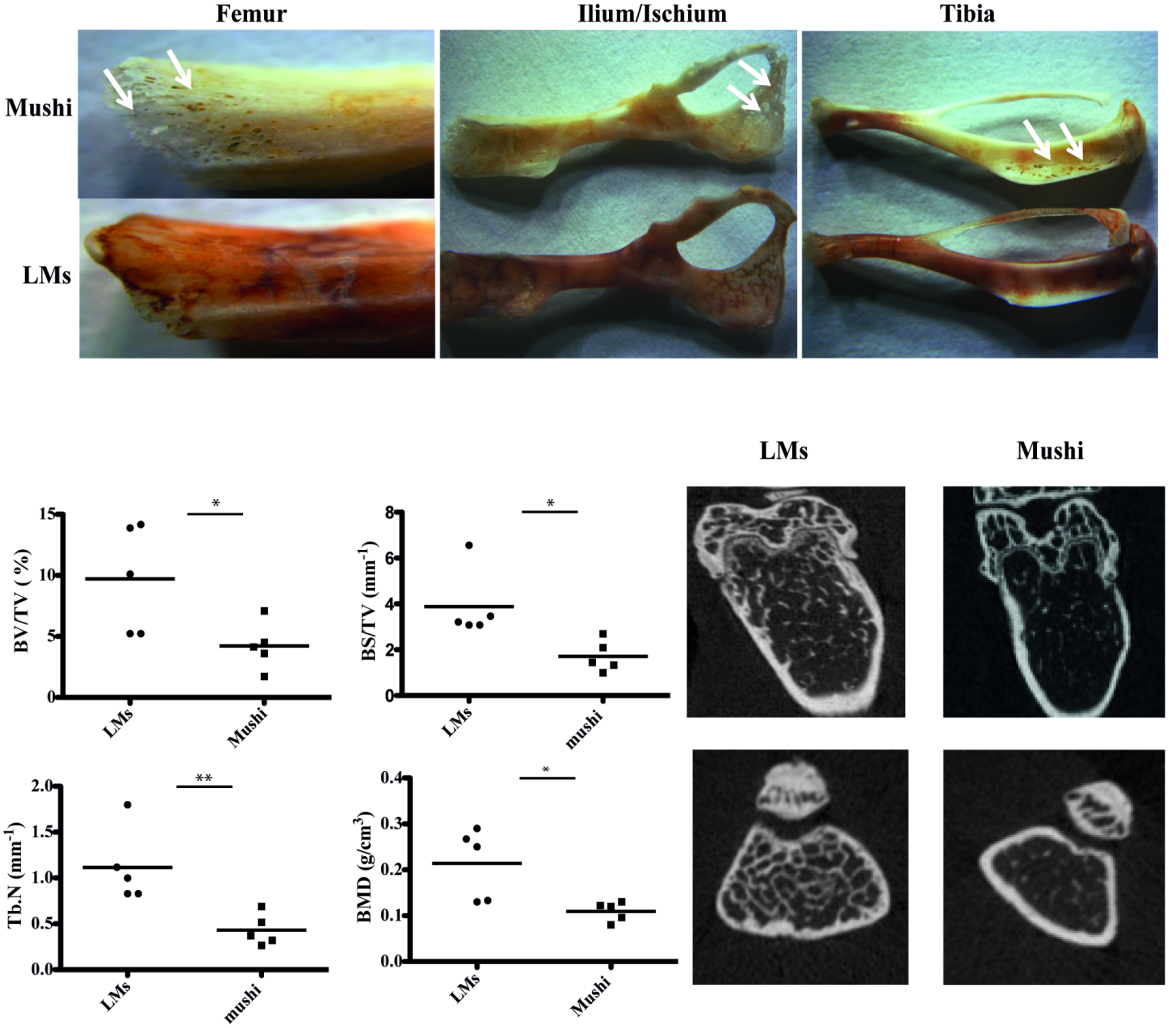
Bone lesions are found in Mushi Tg mice. A) Representative stereo miscroscopic images of 4-month-old littermates (LMs) and sick Mushi mice femur, ilium/ischium and tibia. Arrows indicate the area of bone lesions in Mushi mice. B) Morphometric analyses of LMs and Mushi mice femurs using CT analyzer. Five mice per group, data are from a single experiment representative of two independent experiments. Significance was calculated in relation to wild type group C) Representative axial views of femoral metaphyseal region of LMs and Mushi femoral bone using micro CT scan.

LCH has been shown to be associated with osteolysis. Using our transgenic mouse model for histiocytosis, massive bone lesions were observed in 4 month old mice when DCs started to accumulate. Qualitatively, the lesions appeared well defined in long bones and the cortical bone of the proximal tibial metaphysis was often found completely perforated (Fig 1a). We also observed decreased redness when bone became heavily infiltrated with MuTuDCs due to reduced hematopoiesis and anemia in sick Mushi Tg mice compared to the control healthy Mushi Tg mice (Fig 1a).

To quantify the extent of the bone lesions and the structural parameters, femurs were analyzed with micro-CT. The histomorphometry indicated that Mushi Tg mice had a statistically significant lower percent bone volume, bone surface density, trabecular numbers and bone mineral density in the femur compared to control mice (Fig 1b).

### 2 MuTuDCs infiltrate bone marrow of Mushi mice

**Fig 2:**
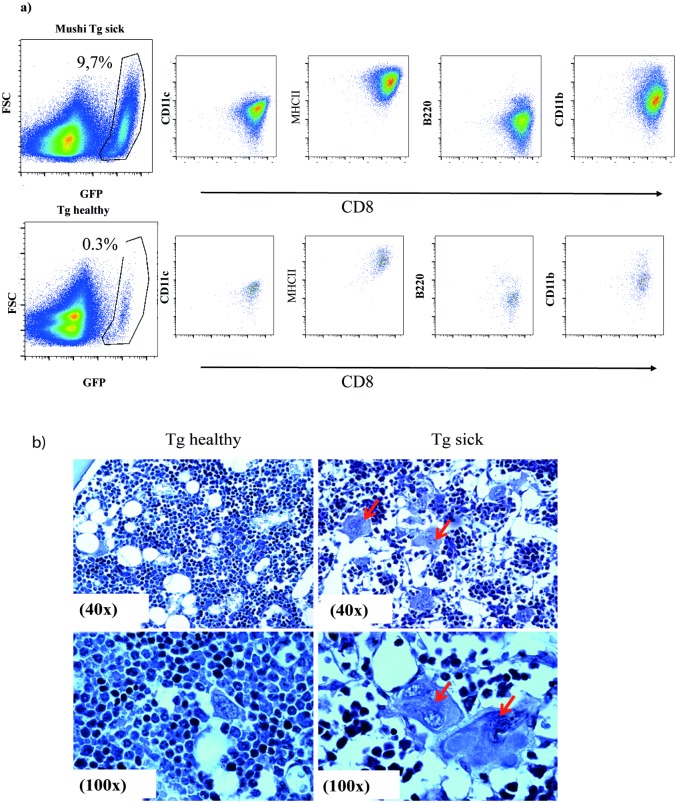
MuTuDCs are recruited to the bone marrow of Mushi mice. A) Flow cytometric analyses of bone marrow from 3 to 4 months old healthy Mushi Tg mice or sick Mushi Tg mice. Representative FACS plots of DC markers showing an extensive cellular infiltrate of GFP positive DCs in the bone marrow of the mice. Data are representative of at least three independent experiments of five mice each. B) Upper panels: H&E staining of paraffin embedded sections of bone marrow from Tg healthy or Tg sick mice (40X). Lower panels: Higher magnification image (100X) shows the presence of classical osteoclast morphology in sick Mushi Tg mice. One representative image from three independent experiments is shown.

As previously described, sick Mushi Tg mice display DC tumorigenesis mainly in spleen and liver but also in bone marrow, thymus and mesenteric lymph nodes [13]. We therefore sought to adapt this mouse model in order to understand how DCs might be involved in the observed bone-resorptive process. Evidence has accumulated indicating that DCs can be influenced by the bone marrow environment to participate in bone resorption through their transdifferentiation into osteoclasts. We addressed the question of whether peripheral DCs were recruited to the tissue surrounding the area of the lesions. FACS monitoring of the GFP positive DCs recruited to the bone marrow showed extensive cellular infiltrates of GFP positive MuTuDCs in sick Mushi Tg mice (Fig 2a). In parallel, histological analyses of the femur from sick Mushi Tg mice revealed a higher number of osteoclasts compared to control healthy Mushi Tg mice (Fig 2b). After adoptive transfer of MuTuDC lines into Rag KO mice, we harvested bones at early time points (when only 1% of BM cells were MuTuDCs) or late (when 38% of BM cells were MuTuDCs). As shown in S1 Fig, more osteoclasts as illustrated by TRAP activity were found in the latter.

We crossed the Mushi Tg mice with Rag KO, perforin KO and Rag/Perforin double KO (DKO) mice. The KO and DKO Mushi Tg mice developed tumors faster but bone lesions were comparable when tumors developed (S1 Fig).

### 3 DCs can differentiate into active osteoclasts in vitro

**Fig 3:**
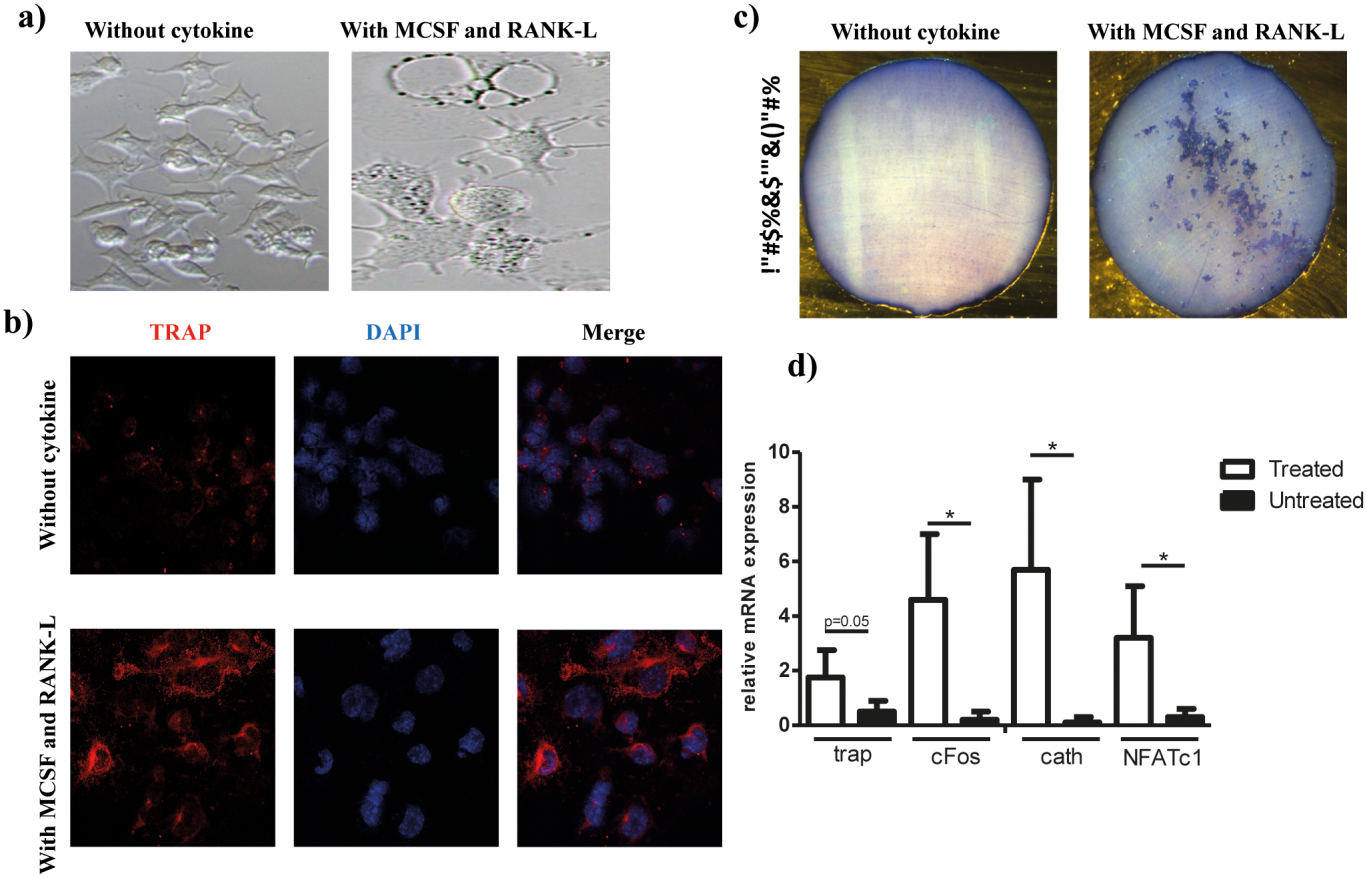
Differentiation of MuTuDCs into functional osteoclasts in vitro. A) Morphology of osteoclasts induced from DCs. MuTuDCs were cultured for 12 days either with or without M-CSF and RANK-L. B) Immunofluorescence analyses for the evaluation of osteoclats differentiation. TRAP-positive cells are stained in red. Nuclei are stained with DAPI (blue). C) Resorption activity was assessed on dentine slices following culture of MuTuDCs with M-CSF and RANK-L. Blue spots indicate the presence of resorption pits for the cytokine-treated cells. All results are representative of two independent experiments performed in three biological replicates. (A-C) One representative image from three independent experiments is shown. D) mRNA relative expression levels of osteoclast specific genes. TRAP, cFos, cathepsin and NFATC1 were measured by qRT-PCR, normalized to β-actin and analyzed with qBasePlus software. The results are representative of 3 independent experiments with three biological replicates per experiment.

In vitro generated DCs or splenic DCs have been shown to form osteoclasts in the presence of RANK-L and M-CSF. We therefore, analyzed the osteoclastogenic potential of our DC line in vitro. Using clonal DC lines formally exclude a role of monocytes or other contaminating cells. As shown in Fig 3, the differentiation of DCs into osteoclasts was highly efficient as, after 10 days in culture, 100% of the cells were TRAP^+^, with more than 80% being multinucleated (Fig 3b). Moreover, these cells generated resorption pits when cultured on dentine slices (Fig 3c), displaying the typical behavior and phenotype of functional osteoclasts.

The expression levels of various osteoclastogenic genes were also monitored by qPCR during MuTuDC differentiation into osteoclasts. Expression levels of TRAP, c-Fos, nFATc1 and cathepsin-K, typical markers of osteoclasts, were significantly up- regulated in MuTuDCs after RANK-L and M-CSF treatment (Fig 3d).

These results confirmed that MuTuDCs have the potential to differentiate in vitro into functional osteoclasts in the presence of M-CSF and RANK-L and in the absence of added IL-17.

### 4 MuTuDCs promote osteolytic lesions

To test the hypothesis that DCs from Mushi Tg mice promote osteolytic lesions, we established a model in which MuTuDCs are injected subcutaneously on the calvaria, close to the left parietal bone. In the other model, the mice received intravenous injections of MuTuDCs. In order to be able to transfer the large T expressing tumor cells into immunocompetent mice, host CD8+ T cells were depleted with an anti-CD8β antibody before adoptive transfer [18]. The MuTuDCs were allowed to proliferate for approximately 4 weeks before the mice were sacrificed. After sacrifice, skulls and hind limbs were collected for the monitoring of osteolysis by stereomicroscopy or CT scan.

**Fig 4:**
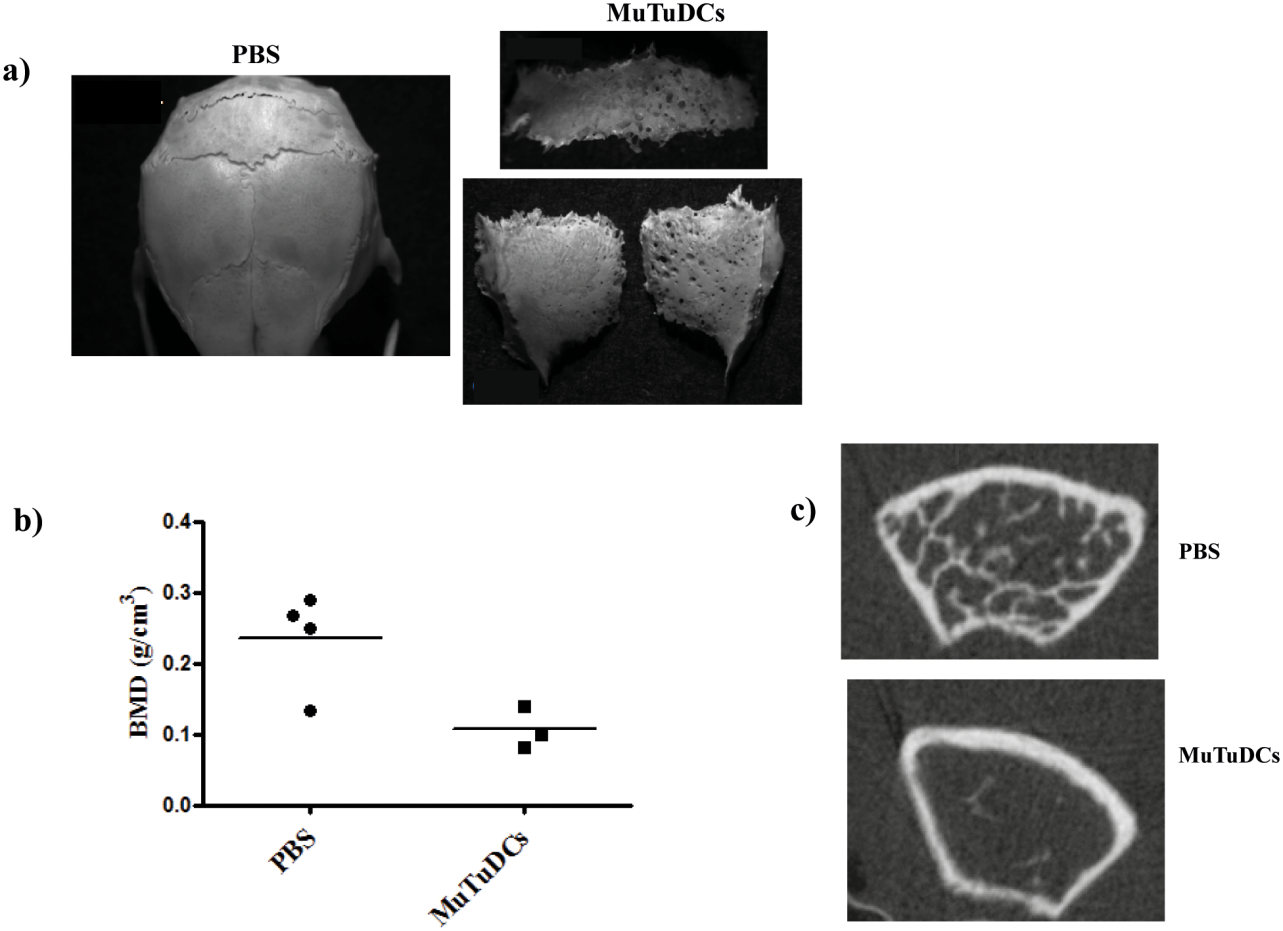
Transfer of MuTuDCs in vivo induces osteolytic lesions. A) Representative stereo microscopic images of skulls from control mice or MuTuDC injected subcutaneously on the calvaria. 4 weeks post-injection, mice that received MuTuDCs had significant osteolytic lesions compared to control mice. The bones became very fragile and disintegrated after isolation. Five mice per group, data are from a single experiment representative of three independent experiments. B) Morphometric analysis of PBS and MuTuDC injected mouse femurs using CT Analyzer one month after intravenous MuTuDC adoptive transfer. The metaphyseal trabecular bone region in the distal femur was examined. Data are representative of two independent experiments of 3–4 mice per group. C) Representative image of axial views of femoral metaphyseal region of control and injected mice using micro-CT scan. Three to four mice were used for each experimental condition.

As shown in Fig 4a, 4 weeks after injection, mice that received MuTuDCs subcutaneously on the calvaria had large osteolytic lesions compared to mice injected with PBS. In addition, the histomorphometry analysis revealed that the mice injected intravenously with MuTuDCs had a statistically lower femur bone mineral density compared to the control mice injected with PBS (Fig 4b). First signs of bone destruction become detectable after 1–2 weeks (data not shown). This indicates that MuTuDCs are able to promote bone lesions.

### 5 Bone lesions are independent on both T and B cells

**Fig 5:**
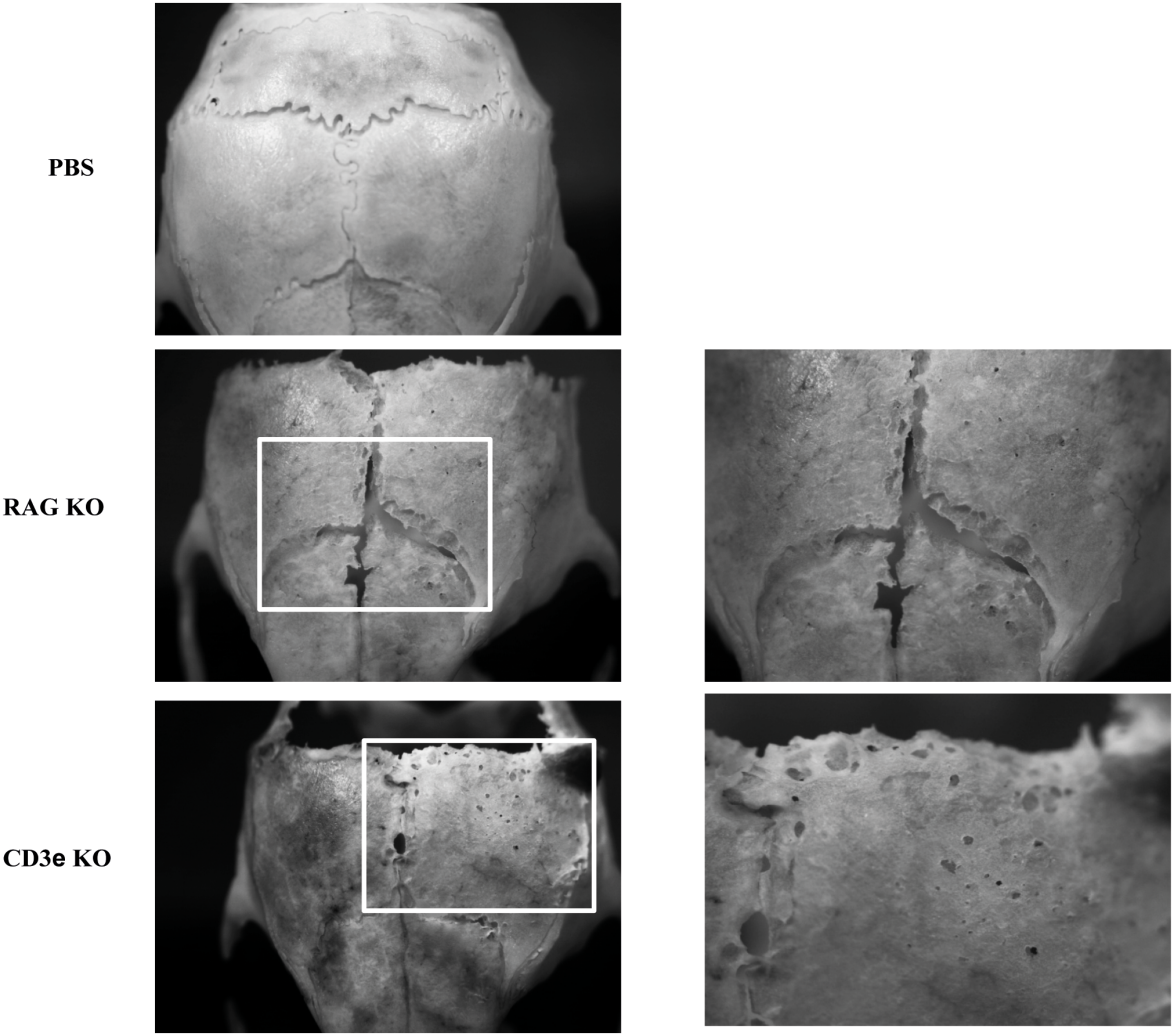
Bone lesions are independent of both T and B cells. Rag KO and CD3ε KO mice were used as recipients of MuTuDCs. Representative of stereomicroscopic images of skulls from wild type, Rag KO and CD3ε KO mice injected subcutaneously with MuTuDCs on the calvaria. Data are from a single experiment representative of two independent experiments. Five mice per group in each experiment.

It has been shown that under inflammatory conditions, T and B lymphocytes contribute to the acceleration of bone resorption as they become significant producers of RANK-L and TNF-α, major cytokines, which regulate osteoclasts differentiation [19,20]. To assess whether T cells or B cells were required to induce bone loss, we challenged our lytic bone lesion model using CD3ε KO or RAG KO recipient mice. Both KO mice, injected with MuTuDCs on the calvaria, did show severe bone lesions (Fig 5). Tumors grew quicker in CD3ε and Rag KO mice. We also produced Mushi x Rag KO and Mushi x CD3εKO mice. These mice show a comparable bone destructions when having comparable bone marrow infiltrations with MuTuDCs (See Fig S2).

### 6 Mice are protected from osteolytic lesions with osteoprotegerin or bisphosphonates but not Etanercept (Enbrel)

**Fig 6:**
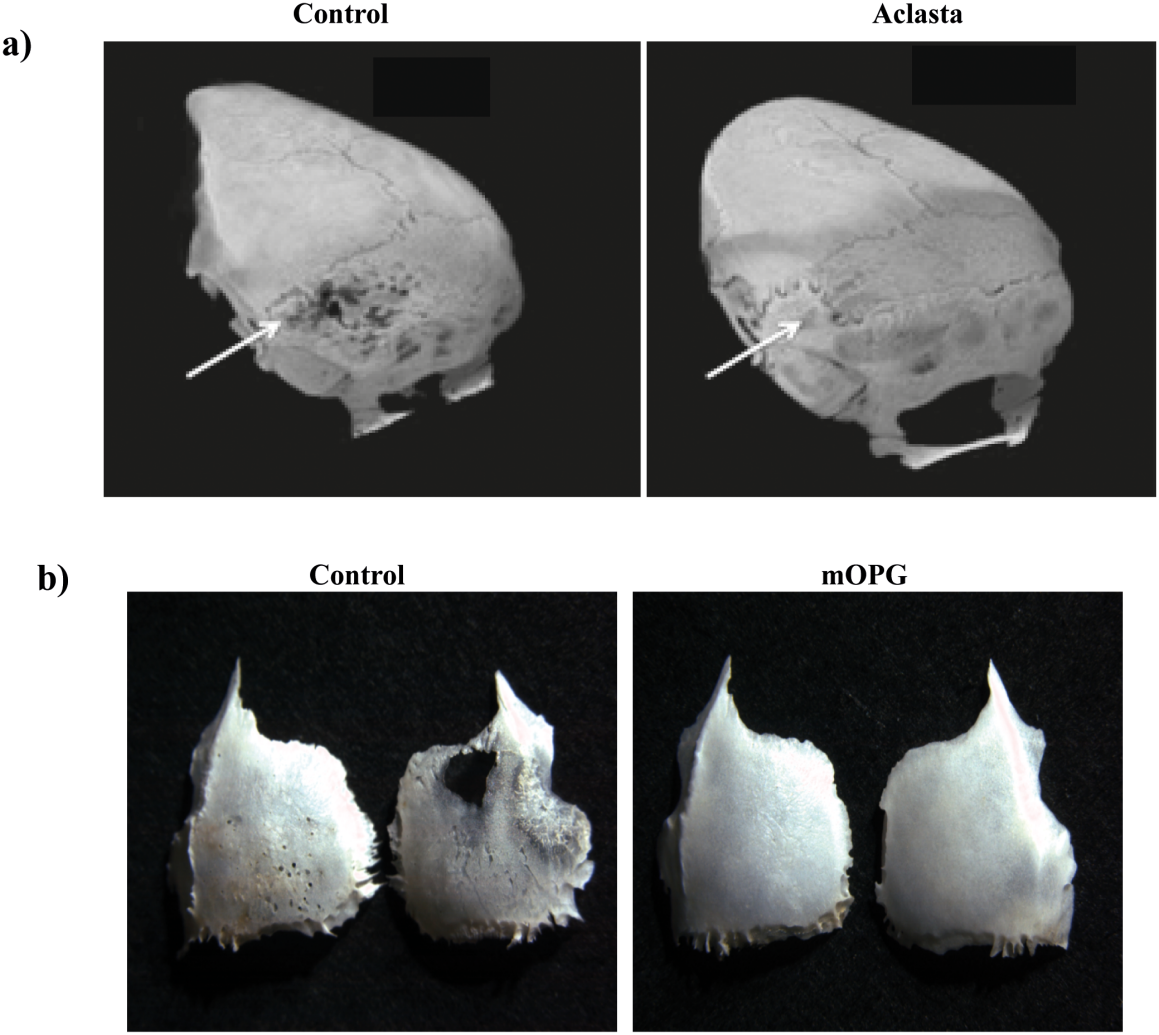
Mice are protected from lytic bone lesions using bisphosphonates and osteoprotegerin (m OPG). A) Representative CT Scan images of skulls of MuTuDC injected mice (subcutaneously on the calvaria, close to the left parietal bone). Mice were treated with either PBS (control) or the biphosphonate Aclasta B) Representative stereomicroscopic images of skull bones of mice injected as above with PBS (control) or mOPG one week following adoptive transfer of MuTuDCs. Images in A and B show the absence of bone lesions after Aclasta or mOPG treatments. Arrow indicates the site of MuTuDC injections. Data are from a single experiment representative of two independent experiments. Five mice per group in each experiment. Similar results were obtained after i.v. injection of MuTuDCs (data not shown).

The beneficial effect of bisphosphonates as anti-resorptive drug in patients with osteoporosis has already been demonstrated [21,22]. To investigate whether the administration of bisphosphonate (Aclasta) would suppress bone lesions after adoptive transfer of MuTuDCs subcutaneously on the calvaria, mice were treated with a single dose of PBS or Aclasta one week after MuTuDC injection. No bone lesions were observed in Aclasta treated mice (Fig 6a).

Osteoprotegerin (OPG), a soluble decoy receptor for RANK-L, is known to be a potent inhibitor of osteoclast differentiation. In order to determine whether mouse (m) mOPG-Fc would inhibit the accumulation of osteoclasts, and thereby protecting mice against lytic bone lesions, we treated mice twice a week with subcutaneous injections of mOPG, starting one week after the transfer of MuTuDCs on the calvaria. As shown in Fig 6b, the mOPG-Fc treatment prevented MuTuDC induced bone loss. Etanercept (Enbrel), a TNFα neutralizing antibody had no effect on the bone lesions (data not shown)

### 7 Bone resorption activity is restored in DCs-treated oc/oc mice

In order to investigate whether the MuTuDC lines can generate osteoclasts in vivo, we have used the already described osteopetrotic oc/oc mouse model to assess the in vivo capacity of DCs to give rise to osteoclasts. These mice have nonfunctional osteoclasts and die at about 3 weeks of age because of their severe osteopetrotic phenotype [23].

**Fig 7:**
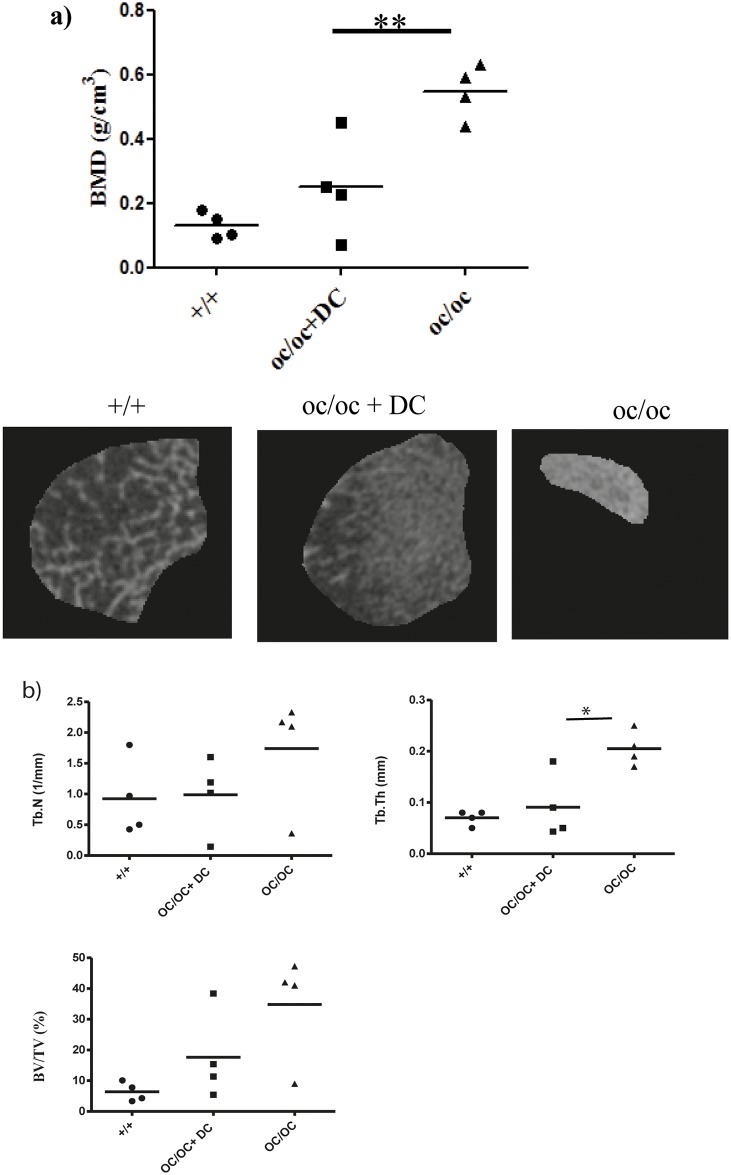
Prevention of the osteopetrotic phenotype in MuTuDC-treated oc/oc mice. A) Bone mineral density analyses of femur using CT Analyzer of normal littermates (+/+), MuTuDC-treated *oc/oc* mice as well as untreated oc/oc mice. The latter was smaller since they did not survive longer than 15–20 days. B) Bone morphometric analysis on control littermates, MuTuDC-treated *oc/oc* mice and untreated oc/oc mice. Four mice per group, data are from a single experiment representative of two independent experiments.

Bone histomorphometry analyses of MuTuDC-treated oc/oc mice, which all survived for at least one month after birth, revealed statistically lower bone mineral density as shown for the femur compared to the untreated control oc/oc mice (Fig 7). This result is in accordance with previous studies using splenic DCs or hematopoietic stem cells [9,24].

## Discussion

We report the first mouse model of LCH-like disease, which exhibits the characteristic symptoms of human histiocytosis including lytic bone lesions, as demonstrated by computed tomography and histological studies. The presence of bone lesions 3–4 months after birth in the Mushi mouse model, along with the other symptoms already described [13], is in line with the defined clinical criteria for the diagnosis of human histiocytosis. In addition, our study demonstrates that DCs are the major cause of bone lesions in our model. We show that adoptive transfer of MuTuDCs induces bone lesions within 1–2 weeks and this effect is independent of T and B cells. Moreover, we show that the administration of bisphosphonate (Aclasta) or mOPG to Mushi Tg mice, but not Etanercept (Enbrel, a TNFα-neutralizing antibody), prevented the apparition of bone lesions. Importantly, we have also shown that injected DCs are able to partially revert the osteopetrotic phenotype of *oc/oc* mice *in vivo*.

A major obstacle to elucidate the pathophysiology of LCH disease is the lack of a reliable animal model. Indeed, the authors of previously described animal models for histicytosis did not report any information related to bone lesions [10,12]. Therefore, the availability of Mushi mice should not only provide the opportunity to conduct prospective and well-controlled studies, but should also allow addressing important questions concerning the pathology of the disease at the cellular and biochemical levels. In this regard, this mouse model represents a powerful tool to develop new therapeutic strategies to prevent and cure bone lesions in human patients.

Bisphosphonates, which are known to inhibit the recruitment of osteoclasts and to reduce their activity, are widely used in the treatment of a variety of bone diseases and recently have become a standard treatment for LCH [25–27]. Our observations corroborate these clinical observations as a single Aclasta injection reduced bone lesions in Mushi mice. This is a very interesting point for further researches in the development and testing of new preclinical drug targets to prevent or cure of bone lesions.

In addition, we found that mOPG treatment prevented bone loss induced by DCs. Osteoprotegerin is known to be a potent inhibitor of osteoclast differentiation. In the context of our mouse model of LCH, we propose that DCs contribute to bone resorption directly through the interaction between RANK-L and its receptor, RANK, commonly expressed by the DCs, a key feature for osteoclasts differentiation. Moreover, treatment of Mushi mice with Etanercept (Enbrel, a TNF-α neutralizing antibody) did not prevent the apparition of lytic bone lesions. (Data not shown)

Due to their capacity to process and present antigen, DCs are the most effective antigen-presenting cells (APCs) for the activation of naive T cells. Recent studies have shown that immature DCs can differentiate into osteoclast-like cells [7–9,28]. In agreement with these observations, our results extend these *in vitro* and *in vivo* data by showing that, not only DCs generated *in vitro* or splenic DCs, but also the MuTuDC lines can efficiently differentiate into osteoclasts in the presence of M-CSF and RANK-L.

Several studies have reported that inflammatory CD4^+^ T cells play a role in the differentiation of DCs into osteoclasts through maintenance of a high level of RANK-L expression in vivo [29–31]. In contrast with these studies, our results indicate that T and B cells are not required for the differentiation of MuTuDCs into osteoclasts. In line with our results, it was also reported that T cells are not required for normal bone homeostasis as T cell–deficient mice were shown to have a normal bone phenotype [32].

In human histiocytosis, the fusion of DCs into multinucleated cells with bone resorption properties has been shown to be dependent on the cytokine IL-17 [3]. In the present study, no role for IL-17 was demonstrated for the differentiation of MuTuDCs into osteoclasts: serum levels at different time points of tumorigenesis, injection of biotinylated anti-IL-17 antibodies for 3 days and quantitating IL-17/antibody complexes in sera by ELISA as well as mRNA quantitation in bone marrow in affected mice by RT-PCR all failed to show any evidence regarding the role of IL-17 (data not shown). In line with our observations, other previous studies were also unable to identify evidence of IL-17A expression in LCH lesions [33,34].

In summary, the data presented here supports the idea that controlling the recruitment of DCs to the bone, or their fusion into giant cells, could be part of a therapeutic approach to limit joint injury in diseases, such as histiocytosis and osteoarthritis. The model described in this study could be a valuable tool to screen (within 1–2 weeks) new agents for the treatment of diseases associated with bone destruction, both in vivo and in vitro, such as histiocytosis, periodontitis and rheumatoid arthritis.

## Supporting Information

S1 Fig TRAP activity during bone lesion development. (TIF)

S2 Fig Survival curves of Mushi Tg mice and various KO mice Tg for Mushi. (TIF)
