# Supplementary figures and images for: Dendritic Cells Cause Bone Lesions in a New Mouse Model of Histiocytosis
Source: PLoS One. 2015 Aug 6;10(8):e0133917. doi: 10.1371/journal.pone.0133917 (PMC4527720; doi:10.1371/journal.pone.0133917)

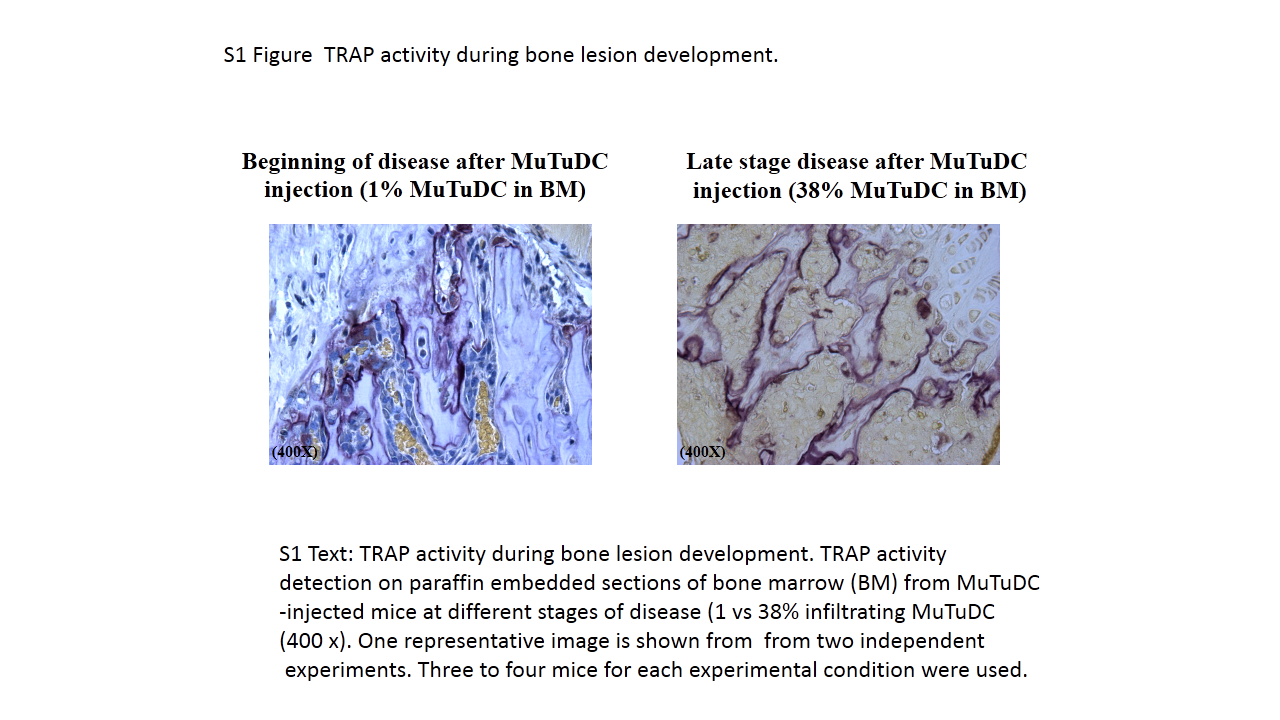

Supplement: S1 Fig — (TIF) [file pone.0133917.s001.tif]

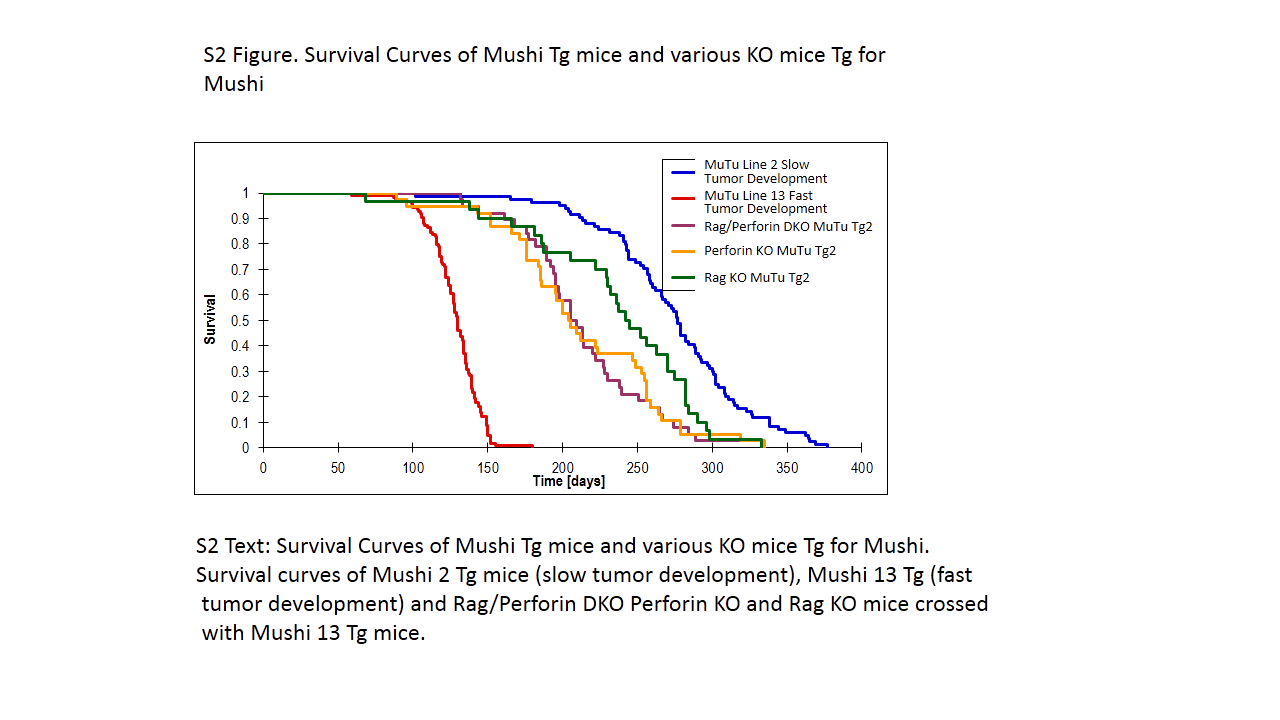

Supplement: S2 Fig — (TIF) [file pone.0133917.s002.tif]
